# Technological frontiers driving new insights in plant RNA biology

**DOI:** 10.3389/fpls.2026.1944001

**Published:** 2026-10-19

**Authors:** Leonardo Furci, Anna M. Klarkowska, Atsushi Shimada, Hidetoshi Saze

**Affiliations:** Plant Epigenetics Unit, Okinawa Institute of Science and Technology Graduate University, Onna, Japan

**Keywords:** enhancers, epitranscriptomics, non-coding RNAs, RNA biology, sequencing technologies

## Abstract

Recent technological innovations in high-throughput sequencing have reshaped our understanding of plant RNA biology, revealing complex regulatory layers beyond primary genetic sequences. In this mini-review, we highlight how emerging methodologies ranging from nascent RNA profiling to direct long-read sequencing have uncovered novel roles for non-coding RNAs, enhancer elements, alternative splicing/polyadenylation, and epitranscriptomic marks in RNA transcriptional control and stress adaptation. For each technique driving these fundamental advances, we provide a brief overview outlining the methodologies and their respective advantages and limitations, guiding the optimal tool for future RNA research. Finally, we propose how integrating these technologies into combinatorial studies will advance a unified, system-wide model of plant RNA dynamics, as well as the potential to resolve co-occurring RNA modifications in the context of an epitranscriptomic “epi-code” governing post-transcriptional fate. Bridging these molecular mechanisms will be crucial for understanding phenotypic plasticity and enabling targeted (epi)transcriptomic engineering to develop climate-resilient crops.

## 1 Introduction

Beyond the central dogma of molecular biology, stipulating linear flow of information from DNA to mRNA to proteins, multiple layers of RNA biology exist to add critical nuance to expand phenotypic complexity in eucaryotic organisms like plants. For instance, (1) non-coding RNAs (ncRNAs) and transcriptional activity at regulatory elements play significant roles in shaping both local and distal chromatin environments, influencing transcriptional competence and modulating gene transcription rates in plant cells (Marquardt et al., 2023). (2) During transcription, nascent RNAs are subject to a variety of chemical modifications that constitute additional layers of functional information, contributing to the diversity of the epitranscriptome within cellular RNA populations (Cerneckis et al., 2024). (3) Nascent transcripts from protein-coding genes by RNA Polymerase II (Pol-II) undergo co- and post-transcriptional processing to become mature messenger RNAs (mRNAs), including 5′ capping, splicing, and polyadenylation. Under stress or developmental cues, structural editing during transcription such as alternative splicing or alternative polyadenylation allows a single gene locus to generate multiple distinct mRNA transcripts and protein isoforms, expanding the coding capacity and regulatory complexity of plant genomes (Marquardt et al., 2023; Wang et al., 2024; Alhabsi et al., 2025). (4) Similarly, under stress conditions, aberrant or transposable element (TE)-derived transcripts can bypass canonical RNA quality control and be hijacked for post-transcriptional gene silencing (PTGS) (Elmayan et al., 2025). (5) Lastly, combinations of different epitranscriptomic modification on mature mRNAs regulate key aspects of RNA metabolism, such as splicing, localization, translation efficiency, and stability, for accurate mRNA fate determination (Shen et al., 2023). These nuances in mRNA modulation are fundamental to sessile plants for the fine-tuning of cellular regulation to achieve phenotypic plasticity from a static genetic code, and successfully thrive under environmental fluctuations.

Over the last two decades, major advances in RNA isolation, chemical processing, and high-throughput sequencing technologies have provided novel insights into these complex facets of RNA biology. In this mini-review, we highlight recent studies leveraging these techniques to uncover the critical functions of RNA processing and modifications, while separately providing a brief overview of the key sequencing techniques driving these discoveries. For each technique, we outline the underlying methodology alongside its respective advantages and limitations, offering a practical guide to help readers select the optimal tool for their own research. Finally, we discuss how these expanding methodologies, especially if combined, will continue to broaden our understanding of how RNA dynamics fine-tune gene expression and mediate plant responses to fluctuating environmental and developmental signals.

## 2 Transcriptional regulation by non-coding RNAs and enhancer elements

Recent studies in the model plant Arabidopsis (*Arabidopsis thaliana*) have uncovered critical roles for ncRNA transcripts in transcriptional regulation. By analyzing publicly available Immunoprecipitation followed by analysis of RNA ends (IPARE; **Box 1**) data (Tsuzuki et al., 2020), Yuan et al. reported that chromatin-bound ncRNAs transcribed by the plant-specific RNA Polymerase V (Pol V) regulate genes involved in plant defense responses (Yuan et al., 2024). Although Pol V is traditionally known for its role in RNA-directed DNA methylation (RdDM), this regulatory phenomenon appears to function independently of the canonical RdDM pathway, as similar functions were not observed for RNA Polymerase IV (Pol IV), another core component of the RdDM machinery (Erdmann and Picard, 2020). Montez et al. using 3′ RNA‐seq (**Box 1**) described another example of ncRNA-mediated transcriptional control involving *PUPPIES*, long non-coding RNAs (lncRNAs) derived from the promoter of the *DOG1* gene, a central regulator of seed dormancy and salt stress responses (Montez et al., 2023). *PUPPIES* enhances *DOG1* expression by promoting promoter-proximal pausing of RNA Polymerase II. A subsequent study identified an additional *DOG1*-associated lncRNA named *MUSHER* (Sacharowski et al., 2025). By utilizing a combination of 3′ RNA-seq and different chromatin immunoprecipitation techniques (ChIRP-seq and ChIDP-seq; **Box 1**) they could uncover that *MUSHER* binds to the *DOG1* locus and modulates alternative polyadenylation by recruiting the cleavage and polyadenylation specificity factor (CPSF) complex (Sacharowski et al., 2025). Interestingly, both *DOG1* and *PUPPIES* are associated with the U1 small nuclear ribonucleoprotein (snRNP), a complex that typically binds to 5′ donor sites of introns to mediate RNA splicing and is essential for the chromatin retention of these lncRNAs. Notably, *COOLAIR*, an antisense lncRNA transcribed from the flowering regulator gene *FLC*, is also retained on chromatin in a U1 snRNP-dependent manner, suggesting a broader, previously unrecognized role for U1 snRNP in the chromatin association of lncRNAs (Sacharowski et al., 2025). These emerging examples underscore the complexity of RNA-based regulation, revealing how non-coding transcripts and RNA processing components intersect with transcriptional control mechanisms in plants.

Box 1 **Brief description of novel sequencing techniques and their application in RNA biology.**

3′-RNA seq: a transcriptomic technique that sequences only the 3′ polyadenylated end of mRNAs. This technique is highly cost-effective for differential gene expression analysis, by reducing the number of reads per sample allowing for deeper sequencing. It also has higher sensitivity than conventional mRNA-seq, making it ideal with lower RNA input starting amounts, such as in the case of non-model plant organisms. However, it is not well suited for detection of alternative splicing or novel isoforms (Krzyszton et al., 2022; Zhang et al., 2022a).

IPARE (Immunoprecipitation followed by analysis of RNA ends): an immunoprecipitation-based variation of NanoPARE-seq, which combines 5′ sequencing of tagmented cDNAs with body sequencing on the same molecule from transposase-inserted adapters, used to accurately profile transcription start sites, noncapped RNA 5' ends, and small RNA targeting events (Schon et al., 2018). In IPARE variant, an upstream immunoprecipitation step against target RNA-binding protein allows for specific sequencing of all RNAs generated or processed by the targeted candidate protein (Tsuzuki et al., 2020).

ChIRP-seq (Chromatin isolation by RNA purification): This method utilizes oligonucleotide probes to affinity-capture target lncRNA:chromatin complexes *in vivo* after crosslinking. Sequencing of immunoprecipitated DNA bound to RNA and RNA-binding proteins allows to map long RNA occupancy across the genome, generating a map of genomic binding sites at a resolution of several hundred bases with high sensitivity and low background (Jathar et al., 2017; Sacharowski et al., 2025).

ATAC-seq (Assay for transposase-accessible chromatin using sequencing): technique that maps genome-wide chromatin accessibility of DNA using the modified transposase Tn5, which simultaneously fragments accessible DNA and tags it by inserting sequencing adapters (tagmentation) (Buenrostro et al., 2013). The detection of intergenic accessible DNA is considered the first hallmark of putative enhancer elements.

csRNA-seq (Capped-small RNA sequencing): precisely maps active transcription start sites (TSS) by selectively isolating or enriching 5'-capped RNAs through enzymes such as Cap-Clip, which allows specific sequencing of nascent short transcripts processed by Pol-II (Duttke et al.). Compared to conventional RNA-seq, it allows detection of both stable mRNAs and highly transient transcripts such as eRNAs.

STARR-seq (Self-Transcribing Active Regulatory Region Sequencing): a sequencing technique to identify candidate genomic regions with enhancer-like activity from arbitrary DNA sources. In plants, sheared fragments of genomic DNA are fused upstream of a minimal 35S promoter paired with reporter gene such as GFP and a barcode. Following the transient transformation of these plasmid libraries into protoplasts or tobacco leaves, cDNAs produced from each vector are sequenced to determine which genomic regions possess the strongest enhancer activity by evaluating the relative expression levels of the reporter gene (Jores et al., 2020). This method has high-throughput screening capabilities and can be used to annotate new enhancer regions. However, the vector library preparation step makes it so the effect of native chromatin structure on enhancer activities cannot be determined using this method.

RAMPAGE (RNA Annotation and Mapping of Promoters for the Analysis of Gene Expression): a variation of conventional RNA seq that specifically enriches for the 5'- ends of complete RNA molecules, allowing to precisely pinpoint the TSS genomic location (Batut and Gingeras, 2013; Beernink et al., 2025). Used by the ENCODE consortium to annotate TSSs in transcriptomes or detect novel transcribed promoter/enhancer elements. Though less indicated than csRNA-seq for the detection of transient RNAs.

GRID-seq (Global RNA interactions with DNA by deep sequencing): this technique uses a biotinylated bivalent linker to directly ligate RNA to DNA following double nuclear fixation with formaldehyde and disuccinimidyl glutarate (DSG). After conversion of the DNA-ligated RNA into cDNA, the cDNA-DNA hybrid fragments are deep-sequenced to map the full spectrum of chromatin-interacting RNAs (including ncRNAs) at their genomic location of contact (Zhou et al., 2019).

CAP-C (Chemical-crosslinking assisted proximity capture): a technique to capture proximal chromatin contacts without formaldehyde prefixing, but utilizing instead poly (amidoamine) dendrimers which crosslinks to double-stranded DNA on UV irradiation (You et al., 2021). Compared to other assays such as Hi-C, lack of crosslinked proteins masking enzymatic digestion sites or hindering proximal ligation of digested DNA this technique allows to obtain chromatin contact maps at sub-kilobase resolution with low background noise, validating putative cis interactions between TSS and distal regulatory elements with increased specificity (You et al., 2021).

GRO-seq (Global run-on sequencing): nuclear run-on techniques allow for specific selection of actively transcribed RNAs, rather than steady-state RNA levels which result from the combination of transcription, post-transcriptional processing, and degradation. In nuclear-run-on, cells or nuclei are incubated with bromouridine (BrU)-UTP to label actively transcribed RNAs, in the presence of transcriptional inhibitor sodium lauroyl sarcosinate (sarkosyl) which prevents attachment of new RNA polymerases on DNA. GRO-seq combines nuclear run-on technique with global sequencing of labelled, newly synthesized transcript to map genome-wide nascent RNAs (Core et al., 2008).

ONT-DRS (Oxford Nanopore technologies Direct RNA Sequencing): a fast-developing third generation sequencing technology using protein nanopores embedded inside an electrically resistant membrane. As RNA molecules pass through the pores, each different nucleotide alters the electrical current in a unique pattern. During basecalling, integrated software interprets the electrical current signal back into sequence letter. As native RNA molecules are directly sequenced through the pores, library preparation does not need complex chemical labeling, optical scanning, or PCR amplification. DRS generates very long reads starting from the 3′ end of the poly-A tail, which in most cases can cover the whole mRNA, making it highly suitable to identify novel isoforms, de novo transcriptome annotation or precise poly-A length quantification. In addition, chemical modifications of RNA bases such as m6A or m5C and more can be directly detected by computational neural network models, based on the divergent electrical current shift patterns compared to unmodified bases (Chen et al., 2025). To date however, only m6A detection by DRS has been compared with experimentally validated modification mapping (Parker et al., 2020), whereas for other modifications the accuracy of the computational models remains low, and no correlative experimental validation has been done yet in plants. Additionally, coverage yield relative to cost is still lower than other short read-based sequencing techniques.

m6A-SAC-seq/GLORI-seq: these methodologies take advantage of enzymatic and/or chemical treatments which selectively modify methylated or unmethylated adenosines to alter the RNA base sequence. These alterations are then detected through downstream sequencing to quantify transcript-level ratio of m6A at single base resolution. For m6a-SAC-seq, the bacterial dimethyltransferases MjDim1 converts m6A sites into cyclic-allyl-m^6^A which, after reverse transcription, generate T/C/G mismatches used to quantify m6A rate versus a control (Ge et al., 2023). This technique works well on canonical DRACH m6A sites but not on non-canonical ones, due to the enzyme affinity for GAC sites. On the other hand, GLORI-seq utilizes glyoxal- and nitrite-based chemical reactions which selectively deaminate unmethylated adenosines to inosine while leaving m6A intact. During reverse transcription deaminated inosines are read as guanosine (G), whereas m6A sites are read as A, giving a ratio of methylated vs unmethylated m6A sites (Shen et al., 2024).

RNA BS-Seq (RNA bisulfite sequencing): m5C Purified RNA is treated with sodium bisulfite, which converts unmodified cytosines to uracil, then converted to cDNA with a specialized kit (to account for uracil-heavy RNA) and deep sequenced. Like DNA BS-seq, bioinformatic analysis is then used to obtain the cytosine-to-thymine conversions and calculate methylation levels at single cytidine resolution at precise genomic location (Chen et al., 2022). One disadvantage of this technique is that the bisulfite conversion can cause excessive RNA degradation affecting downstream alignment to transcriptome, or incomplete conversions resulting in false positive m5C hits.

UHPLC-MRM-MS/MS (Ultra-High-Performance Liquid Chromatography coupled with Multiple Reaction Monitoring Tandem Mass Spectrometry): while not a sequencing technique, this is considered the most accurate for targeted quantitative analysis of nucleotide modifications. After extraction, total RNA (or DNA) is digested into single nucleotides and run through a reversed-phase C18 column to separate 5mC from other unmodified ribonucleosides. Then, the ratio of modified/unmodified cytidines is quantified utilizing a triple quadrupole mass spectrometer. As RNA is digested into single nucleotides, this technique provides very accurate global absolute quantification of RNA modifications but loses all information on the transcript/genomic location in which the modification is present or absent (Boulias and Greer, 2021).

M6A-seq (also known as Methylated RNA Immunoprecipitation Sequencing, or MeRIP-Seq): a technique based on antibody-mediated capture of A-methylated RNAs followed by deep sequencing, resulting in enriched peaks of reads around each m6A site (Dominissini et al., 2013). Before the advent of ONT-DRS, this technique was the widely used standard approach for RNA modification analysis, used across multiple organisms. While it has been widely used to study m6A, its use for different RNA modifications has been limited by the sensitivity of the immunoprecipitating antibody, which in some cases has been shown to bind aspecifically creating false positives (Helm et al., 2019).

ChIRP-MS (Comprehensive identification of RNA-binding proteins by mass spectrometry): A variation of Immunoprecipitation-Mass Spectrometry (IP-MS), which combines antibody-based immunoprecipitation with mass-spectrometry proteomics on interacting peptides for high-throughput identification of protein-protein interaction. In ChIRP-MS, biotinylated DNA oligonucleotides are used to capture specific non-coding RNAs and their associated proteins, which are then identified through MS (Chu et al., 2015).

FA-CLIP (Formaldehyde-crosslinking and immunoprecipitation sequencing): sequencing method designed to map RNA-protein interactions at single-nucleotide resolution that utilizes antibody-based precipitation of an RNA binding proteins of interests followed by deep sequencing of the bound RNA. The use of formaldehyde for crosslinking proteins and RNA has been found to be more efficient in plant tissues compared to other methods. This technique can be used to precisely map binding sites of RNA-binding proteins on RNA molecules such as m6A readers (Wei et al., 2018).

Ribo-seq (Ribosome profiling): a technique that allows to obtain a snapshot of all transcripts actively translated at a given state or condition. Before RNA extraction, cells are treated with drugs that halt translation and freeze ribosomes. Then, cell lysate is treated RNase enzymes that degrades unprotected RNA, leaving only the fragments covered by the ribosomes (~30-nucleotide long)as a molecular footprint of ribosomes actively translating mRNAs, which are then deep-sequenced to quantify the relative occupancy of ribosomes for each transcript (Ingolia et al., 2009). When paired with conventional transcriptomics such as RNA-seq, which measures the total mRNA at the steady state in both nucleus and cytoplasm, ribo-seq could be used to infer the translation rate of each transcript by the ratio of its total mRNA abundance (RNA-seq) versus the ribosome-protected fragment abundance (Ribo-seq) (Gorochowski et al., 2019). Additional experimental validations through translatomic or proteomic approaches are still needed to accurately quantify translation rate of each transcript, which are affected by a combination of factors beyond simple ribosome occupancy such as translation initiation, elongation, codon usage, transcript abundance and experimental conditions.

Mammalian enhancer RNAs (eRNAs) are typically 5′-capped, non-polyadenylated, and non-coding transcripts between 50 and 200 bp long which are transcribed unidirectionally or bidirectionally by RNA polymerase II from active enhancers elements. They have been shown to be able to act in *cis* (on the same chromosome), by promoting distal enhancer-promoter chromatin association via interaction with the Mediator complex and recruitment of key transcription factors (**Figure 1**), as well as in *trans* (between different chromosomes) (Arnold et al., 2020). The concept of enhancer-mediated transcriptional regulation has steadily been taking root in the field of plant epigenetics. Multiple studies have shown the presence of enhancer elements in Arabidopsis, rice (*Oryza sativa*), maize (*Zea mais*), and wheat (*Triticum aestivum*) among others, most of which also found transcription events in enhancer loci, producing putative eRNAs (Beernink et al., 2025). However, while several studies strongly suggest that plants enhancer loci can produce eRNAs, the functionality, origin and characteristics of these transcripts to date remain debated. Earlier works based on analysis of nascent transcripts and DNA/chromatin features struggled to detect eRNAs, likely due to lower read coverage and lower levels of bidirectional transcription compared to mammals (Hetzel et al., 2016; Oka et al., 2017). Two studies in Arabidopsis, maize and Cassava (*Manihot esculenta*) described limited divergent transcription associated with enhancer loci resulting in bidirectional transcription (Thieffry et al., 2020; Lozano et al., 2021), while others provided additional evidence of bidirectional eRNA in Arabidopsis (Zhang et al., 2022c; Tremblay et al., 2024). Of these, Zhang et al. combined Assay for Transposase-Accessible Chromatin with sequencing (ATAC-seq; **Box 1**) chromatin accessibility data with canonical RNA-seq on rRNA-depleted RNA to detect ~500 enhancers producing eRNA, 65% of which were bidirectionally transcribed (Zhang et al., 2022c). Interestingly, six functionally validated enhancers also showed transcriptional upregulation during basal immunity activation, unfolding the putative role of plant enhancers in modulating stress responses through transcriptional reprogramming (Zhang et al., 2022c). Tremblay et al. also combined capped-small RNA sequencing (csRNA-seq; **Box 1**) with ATAC-seq and putatively identified 1891 uni- and bidirectional enhancers active in germination (Tremblay et al., 2024). They also identified 381 bidirectional non-coding promoters exhibiting patterns of activity similar to canonical enhancers, suggesting that sense and antisense transcription at enhancer or enhancer-like promoter elements could be mutually exclusive (Tremblay et al., 2024). Application of Self-Transcribing Active Regulatory Region sequencing (STARR-seq; **Box 1**) in Arabidopsis identified 4327 enhancers with various combinations of epigenetic modifications distinctively different from animal enhancers (Tan et al., 2023). Interestingly, very little overlap was found between enhancers regions identified by different strategies, indicating complementarity between methodologies and enhancer types (Tan et al., 2023). Modeling the classification of maize enhancers after mammalian ones, two studies utilized a combination of ChIP-seq histone mark profiling with STARR-seq and RNA Annotation and Mapping of Promoters for the Analysis of Gene Expression (RAMPAGE; **Box 1**), revealing a vast prevalence of bidirectionally transcribed regulatory regions with canonical enhancer characteristics, such as bidirectional transcription and co-occurrence of H3K27ac and H3K4me3 marks (Ricci et al., 2019; Cahn et al., 2024). On the other hand, a subsequent study using csRNA-seq in maize, which focuses on capped nascent RNAs processed specifically by Pol-II, found that regions that initiate stable transcripts, but not those marked by unstable or bidirectional unstable transcripts, show stronger enhancer activity (McDonald et al., 2024). In this study unstable bidirectional transcripts were found to be relatively rare in plants, and the authors argued that most putative enhancer loci producing eRNA in plants could be in fact promoters of misannotated genes, blurring the distinction between promoter and enhancer elements (McDonald et al., 2024).

**Figure 1:**
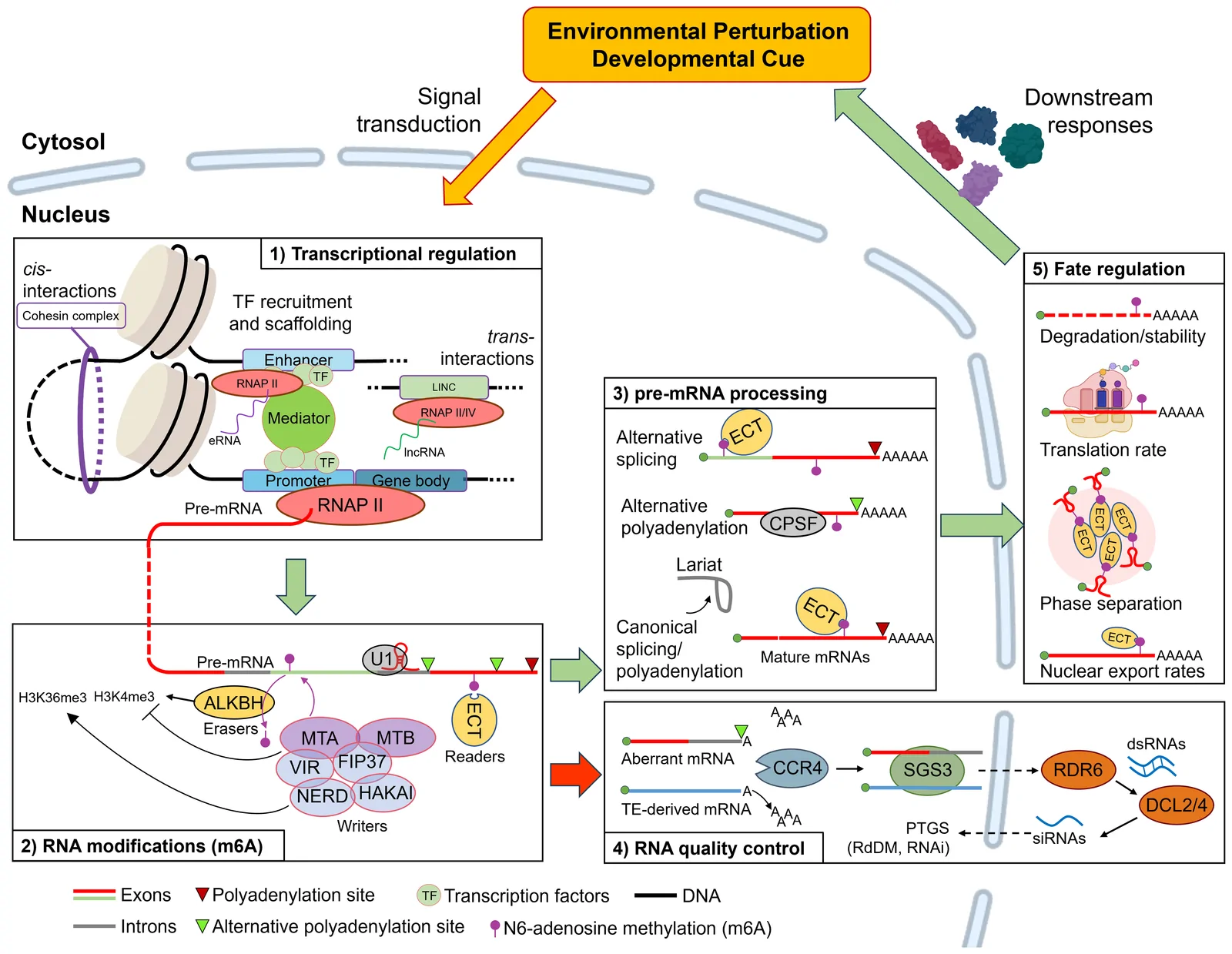
Mechanistic overview of different aspects of RNA biology explored in the text.

The question of whether eRNAs in plants can themselves act in *cis* or in *trans* remains unanswered. A Global RNA Interactions with DNA by deep sequencing (GRID-seq; **Box 1**) study in Arabidopsis showed evidence of 494 lncRNA-DNA interactions, 24% of which were distal (>1kb) and intrachromosomal, 18% interchromosomal, but it remains unclear whether a portion of these interactions could constitute *trans* eRNA activity (Li et al., 2021). Later, a Capture-C assay (CAP-C; **Box 1**) in Arabidopsis identified promoter-promoter and enhancer-promoter interactions, including activity in the previously established COOLAIR enhancer (Zhang et al., 2024). Of the enhancer-promoter interactions 89% were distal, and for some of these, enhancer expression was supported by Global Run-On sequencing (GRO-seq; **Box 1**) data (Zhu et al., 2018), which could suggest *cis* eRNA activity. Interestingly, of the identified promoter-promoter interactions, 77% were intrachromosomal and 23% occurred in *trans* (Zhang et al., 2024), but no data on *trans* interactions were reported for enhancer-promoter pairs, leaving the mechanism for putative eRNA-mediated regulation unclear. It must be noted that, in plants, nascent RNA sequencing techniques such as GRO-seq can detect transcripts from both canonical Pol-II and non-canonical Pol-IV and Pol-V from the RdDM pathway, which are not present in mammals, adding another layer of complexity on the putative origin of eRNAs in plants and their epigenetic regulation.

A key issue with identification of plant enhancers is the lack of consensus on how they should be defined. Applying mammalian “canonical” criteria results in identification of some enhancers (Ricci et al., 2019; Cahn et al., 2024), but not others, as shown by multiple studies in Arabidopsis not observing presence of classical histone modifications or prevalence of bidirectional transcription. Whether plant enhancers should be defined by sharing all canonical characteristics of mammalian enhancers, or primarily by their regulatory function, remains actively debated. The wide diversity of available competing methodologies for detecting enhancers and enhancer transcripts, ranging from transcriptional activity assays to chromatin accessibility and architecture approaches, further complicates their direct identification. Additionally, transcriptional by-products, transient promoter-associated transcripts and, particularly for non-model organism, mis- or unannotated gene transcripts appearing in transcriptomic data could be falsely identified as putative enhancer candidates. Here, we propose a list of possible experimental criteria to help distinguish all transcriptional events with potential enhancer activity from false positives in **Table 1**.

**Table 1:** Possible criteria to distinguish diverse transcriptional events with potential enhancer activity from false positives.

Transcriptional event	Supporting evidence	Refuting evidence (false positive)
Functional eRNA	Gene expression changes from perturbation of eRNA locus; rescue effects	No functional effects of RNA depletion
Transcriptional by-products	Presence of regulatory effects	Weak/no perturbation effects on phenotype, weak reproducibility
Promoter-associated transcripts	Independent enhancer activity	Canonical promoter marks, TSS structure
Unannotated protein-coding transcripts	Lack of coding evidence	Presence of ORFs, ribosome occupancy

While the presented studies show promising candidate enhancers, these findings should be interpreted cautiously as few enhancers in plants have been validated *in vivo*. Some common candidate validation methods include reporter assays, such as GUS (Jores et al., 2020; Tan et al., 2023) or dual luciferase assays (Jores et al., 2024); high-throughput reporter assays (e.g. the aforementioned STARR-seq or Plant STARR-seq (Tan et al., 2023; Jores et al., 2024)); and deletion of regions of interest via CRISPR/Cas9 (Zhao et al., 2022). The latter approach provides the strongest evidence for enhancer function by demonstrating regulatory effects in the native genomic context, whereas reporter-based assays remove candidates from their endogenous environments, crucially the chromatin context, and must be supplemented by further evidence to confirm validity of an enhancer. Even with established plant enhancer loci, the need to disentangle eRNA effects from that of an enhancer itself or from potential RNA interference (RNAi) or RdDM pathways activation, and account for rapid degradation of exosome-sensitive antisense transcripts (in Arabidopsis (Thieffry et al., 2020)), makes plant eRNA validation a major challenge.

## 3 RNA modifications N6-methyladenosine and 5-methylcytidine and their biological function

RNA molecules are decorated with chemical modifications which provide an additional layer of information beyond the one coded by the genetic sequence. The study of the biological role and distribution of these modifications comprises the field of epitranscriptomics (Cerneckis et al., 2024). The most abundant RNA modification is the addition of a methyl group to different carbon or nitrogen positions in the backbone of RNA nucleosides. In plants, two of the best characterized methylated modifications of mRNA are N6-methyladenosine (m6A) and 5-methylcytidine (m5C). These modifications are dynamically modulated through a combination of RNA methyltransferase (“writers”) and demethylases (“erasers”). In Arabidopsis, m6A writers consist of a complex of methyltransferases MTA and MTB with cofactors VIR, E3 ubiquitin ligase HAKAI and FIP37 (Wong et al., 2023). Additionally, two HAKAI-interacting zinc finger (HIZ) proteins HIZ1 and HIZ2 negatively or positively regulate HAKAI-dependent m6A deposition respectively (Zhang et al., 2022b). Recently, plant-specific NERD protein was characterized as an additional component of m6A core writer complex, stabilizing MTA and MTB and interacting with H3K36me3 methyltransferase SDG8 for the transcriptional inhibition at *FLC* locus (**Figure 1**) (Shao et al., 2025). M6A is deposited primarily (but not exclusively) at the DRACH/RRACH (D=A/U/C, R = A/G, H = A/U/C) consensus motif (Shinde et al., 2023). Recent analyses of m6A distribution in Arabidopsis using Oxford Nanopore Technologies Direct RNA Sequencing (ONT-DRS; **Box 1**) confirmed m6A presence in both UTRs and CDS and the highest abundance at 3′-UTRs near the stop codon (Parker et al., 2020; Wang et al., 2024), later demonstrated to be caused by m6A deposition specifically at one nucleotide downstream of CDS’ stop-codon (Furci et al., 2024). By directly sequencing long mRNA molecules, ONT-DRS techniques allow to obtain transcript-level RNA modification quantification at single base resolution. For m6A, ONT-DRS successfully identified experimentally validated DRACH and non-canonical m6A sites (Parker et al., 2020; Wang et al., 2024). Alternative methodologies to obtain transcript-level information of m6A sites is to utilize sequencing techniques that apply chemical modifications which alter the sequencing output of m6A-modified bases compared to unmodified ones, such as GLORI-seq or m6A-sac-seq (**Box 1**). These techniques could also be combined with ONT RNA sequencing following reverse transcription, instead of short read-based techniques, to obtain full-length sequencing of RNA molecules for better mapping accuracy and isoform determination.

For m5C, TRM4A and TRM4B have been identified in Arabidopsis as putative m5C writers in both tRNA and mRNA, of which TRM4B showed the strongest activity by utilizing RNA bisulfite sequencing (**Box 1**) (David et al., 2017), whereas in rice the major m5C methyltransferase is likely OsNSUN2, as observed by quantifying m5C levels in its knock-out mutant by Ultra-High-Performance Liquid Chromatography coupled with Multiple Reaction Monitoring Tandem Mass Spectrometry (UHPLC-MRM-MS/MS; **Box 1**) (Tang et al., 2020). It must be noted that while mass spectrometry-based methods provide very precise stoichiometry for RNA modifications, they cannot determine how modifications are distributed across individual transcripts. Removal of m6A is mediated by ALKBH eraser proteins such as ALKBH9B and ALKBH10B, which remove the methyl group through oxidative demethylation (**Figure 1**) (Han et al., 2023). On the other hand, RNA m5C erasers have not been characterized yet in any plant species. While in animal mRNA m5C is removed by TET-dependent oxidation, plants do not possess TET enzymes. Active DNA demethylation in plants is achieved through m5Cs excision by glycosylase-lyases which require nucleic acid duplexes (Du et al., 2023), hence unlikely to target RNA, still raising questions on whether plants achieve RNA m5C de-methylation through ALKBH family proteins or a completely uncharacterized set of enzymes.

In plants, regulation of mRNA methylation is crucial for transcriptional reprogramming to achieve phenotypic plasticity and acclimation in response to (a)biotic stresses, which plants cannot avoid as sessile organisms. Several studies revealed a clear link between environmental cues such as salt, cold or heat stresses and global changes of m6A and m5C levels at mRNAs affecting transcript abundance, whereas genetic mutants with constitutively altered m6A and m5C levels across their transcriptomes showed physiological changes in their responses to abiotic stresses (Cai et al., 2025). Recently, two studies also addressed the role and dynamics of m6A in pathogen resistance in Arabidopsis using either m6A-seq (**Box 1**) (Prall et al., 2023) or ONT-DRS (Furci et al., 2024). Furthermore, a synonymous mutation affecting an m6A site in the ACC SYNTHASE 2 (ACS2) gene has been implicated in fruit length domestication in cucumber (Xin et al., 2025), underscoring the agronomic relevance of m6A regulation for crop yield and disease resistance.

## 4 Pre-mRNA processing: splicing, alternative polyadenylation and poly(A) tail length regulation

Recent studies have uncovered unexpected roles for RNA regulators traditionally associated with post-transcriptional processing, such as splicing and RNA degradation, in the control of transcriptional events. For instance, the RNA helicase HUA ENHANCER 2 (HEN2), a component of the RNA exosome, facilitates transcription elongation under cold stress by displacing prematurely terminated transcripts from chromatin, as identified using a combination of conventional RNA-seq and nascent RNA isolation by Pol-II immunoprecipitation (Bhat et al., 2024).

Regulation of polyadenylation site (PAS) choice is a key mechanism contributing to transcript isoform diversity and mRNA stability. The RNA splicing factor U1 snRNP has been shown to interact with the transcription termination machinery, including the Cleavage and Polyadenylation Specificity Factor (CPSF) complex, thereby regulating alternative polyadenylation (APA) site selection, by performing U1-specific Comprehensive identification of RNA binding proteins by mass spectrometry (ChIRP-MS; **Box 1**) (Mangilet et al., 2024). In Arabidopsis, global shifts in PAS usage have been observed in response to environmental stimuli such as hypoxia, light, and heat by using variations of 3′-end RNA sequencing (de Lorenzo et al., 2017; Kubaczka et al., 2024; Yu et al., 2024) (**Figure 1**), as well as in response to pathogen attack in rice (Liu et al., 2024). Although technical challenges previously limited the genome-wide identification of PAS, recent advances in Nanopore-based technology now enable transcript-level resolution of polyadenylation dynamics. ONT-DRS has successfully identified previously unannotated PAS in a range of chromatin- and RNA modification-related mutants in Arabidopsis (Berthelier et al., 2023). Additionally, ONT-DRS has been used to map transcriptome-wide changes in PAS under salt stress in maize roots (He et al., 2024). Lastly, ONT platforms allow direct or cDNA-based measurement of poly(A) tail length. Using these methods, dynamic regulation of poly(A) tail length has been observed during developmental transitions in Arabidopsis and maize (Jia et al., 2022; Xu et al., 2024). Together, these findings highlight the complex and dynamic regulation of PAS usage and poly(A) tail length in plant transcriptomes. They underscore the importance of RNA processing in shaping transcriptome diversity, regulating gene expression, and maintaining genome integrity.

## 5 Recent insights in alternative RNA quality control

In plants, aberrant RNAs derived from mis-transcribed protein-coding genes or transcribed TE-genes are usually degraded by the nuclear and cytosolic RNA quality control (RQC) pathways, which include among others nonsense-mediated RNA decay. Under certain biotic or abiotic stresses RQC is impaired and aberrant RNAs are converted through RNA-DEPENDENT RNA POLYMERASE 6 (RDR6) into small interfering RNAs (siRNA) that trigger both transcriptional gene silencing in the nucleus (through the RdDM pathway) and post-transcriptional gene silencing (PTGS) in the cytosol (through the RNAi pathway). Notably, poly(A) tail trimming of aberrant or TE-derived transcripts by the deadenylase CARBON CATABOLITE REPRESSION 4 (CCR4) is required for the processing of RNA substrates by RDR6 into double-stranded RNAs and then siRNAs (Shimada and Saze, 2024). This key step in siRNAs biogenesis also involves the recently discovered RNA-binding protein SUPPRESSOR OF GENE SILENCING3 (SGS3), shuttling siRNA precursors between the cytosol and the nucleus before RDR6 processing into double-stranded RNAs (**Figure 1**) (Elmayan et al., 2025).

## 6 Mature mRNA fate determination

The YT521-B homology domain family proteins EVOLUTIONARILY CONSERVED C-TERMINAL REGION (ECT)1–11 bind m6A-modified mRNA and act as m6A “readers”, regulating post-transcriptional processes such as alternative splicing, alternative polyadenylation, mRNA stability, localization, folding and translation (**Figure 1**) (Nguyen and Kang, 2024). For instance, ECT1 forms cytoplasmic condensates and promotes the decay of m6A-modified mRNA from salicylic acid-responsive genes under salicylic acid treatment, thereby preventing excessive immune activation (Lee et al., 2024), and co-condensing with ECT9 (Wang et al., 2023). Similarly, ECT8 function in promoting degradation of negative regulators involved in abiotic stress responses has been characterized using a combination of m6A-seq, Formaldehyde-crosslinking and immunoprecipitation sequencing (FA-CLIP; **Box 1**) and ribosome profiling (ribo-seq; **Box 1**) (Cai et al., 2024). On the other hand, similar techniques applied to ECT2/3/4 have shown that these readers promote instead stability of their target mRNA (Song et al., 2023). This apparently antagonistic function of m6A in promoting both mRNA stability and decay raises the question of how different m6A readers achieve target specificity to ensure correct post-transcriptional fate of each mRNA molecule. One hypothesis is that m6A readers might be capable of binding to and distinguishing different epitranscriptomic modifications co-occurring simultaneously on the same mRNA molecules. A recent study in mammalian systems confirmed co-occurrence of both m6A and m5C on individual mRNA molecules as close as 5-15nt from each other using ONT-DRS (Acera Mateos et al., 2024), whereas mammalian m5C reader protein ALYREF was found to co-localize with m6A-modified mRNAs in neurons (Orji et al., 2025). This hypothesis would imply an RNA epi-code complementing histone code and DNA methylation, in which epigenetic information determining post-transcriptional fate of each mRNA molecules is encoded by a combination of different epitranscriptomic marks. In Arabidopsis, mRNA export proteins ALY (homologs of mammalian ALYREF) have been shown to preferentially bind single-stranded 5mC-methylated RNA, suggesting a potential role as m5C readers (Pfaff et al., 2018).

## 7 Discussion

In this mini-review, we have summarized recent advances in different aspects of RNA biology, made possible by the innovative sequencing techniques listed/explored. The next major frontier will be to deploy these methods collectively in future combinatorial studies that expose plants to multiple stresses simultaneously. Because such multi-stress, integrated analyses have yet to be conducted, either at the single-gene or whole-transcriptome level, these future efforts will help move closer to a unified, system-wide model of plant RNA dynamics in simulated real-world environments.

In the case of RNA modifications, chemical analysis identified many more in addition to m6A and m5C decades ago, which include for instance 1-methyladenosine (m1A), 7-methylguanosine (m7G), 3-methyluridine (3mU) and 5-methyluridine (m5U), as well as alternative nucleobases like pseudouridine (ψ). To date, the biological function (and, for some, even the presence) of most of these modifications in plants remains completely unknown. Just recently, a study characterized the dynamics and function of m1A in Arabidopsis (Ke et al., 2026). With advances in both immunoprecipitation-based and Nanopore-based sequencing technologies, future studies should aim to dissect not only the dynamics and functions of these modifications independently, but more importantly how they combinatorially interact on the same RNA molecule to create a unique molecular epi-code governing post-transcriptional RNA fate.

Ultimately, bridging the gap between transcriptional regulation, non-coding regulatory networks and RNA modifications into a unified model of their role on the RNA life cycle will transform our understanding of plant phenotypic plasticity, paving the way for targeted (epi)transcriptomic engineering towards novel climate-resilient crops.
